# Fired Bricks: CO_2_ Emission and Food Insecurity

**DOI:** 10.1002/gch2.201700115

**Published:** 2018-04-14

**Authors:** Arun Jyoti Nath, Rattan Lal, Ashesh Kumar Das

**Affiliations:** ^1^ Department of Ecology and Environmental Science Assam University Silchar 788011 India; ^2^ Carbon Management and Sequestration Center Ohio State University Columbus OH 43210 USA

**Keywords:** agricultural production, land degradation, nutritional security, soil quality

## Abstract

Fired bricks are used for construction purposes over the millennia, going back to the Indus Valley Civilization. The traditional brick‐making process involves removal of agriculturally productive topsoil rich in clay and soil organic matter contents. In addition to the removal of the fertile topsoil and accelerated degradation by other processes, the traditional clay brick making process also emits CO_2_ and other gases into the atmosphere. Therefore, the present study aims to assess the impact of brick making in India on: (i) the magnitude of annual CO_2_ emission and (ii) the loss of agricultural production. Currently, 0.7 Mha (million hectare) of agricultural land is under brick kilns that produce ≈250 billion bricks annually. It is estimated that soil organic carbon lost through the firing process of 250 billion bricks is 5.58–6.12 Tg (teragram) (20.48–22.46 Tg CO_2_), and in conjunction with clay burning and coal combustion the process releases 40.65–42.64 Tg CO_2_ into the atmosphere per annum. Brick kiln also impacts quality of the exposed subsoil, and may also reduce 60–90% agronomic yield. Therefore, brick making from topsoil exacerbates food and nutritional insecurity by degrading soil quality, and increases risks of climate change through increase in gaseous emissions.

The agriculture and allied sector continues to be pivotal to the sustainable growth and development of the Indian economy. Not only does it meet the food and nutritional requirements of 1.3 billion people in India but also contributes significantly to livelihood security and employment generation.^1^ However, soils of India are prone to a range of degradation processes and as much as 114 million hectares (Mha) are affected by degradation including 24 Mha by water erosion, 9 Mha from wind erosion, 23 Mha by chemical degradation, 47 Mha by physical degradation, and 12 Mha by a combination of processes.^2^ Presently, India supports about 17.4% of the world's human and 15% of livestock population on merely 2% of global geographical area and 1.5% of global lands under forest.^2^ The per capita availability of agricultural land has declined from 0.48 ha in 1951 to 0.14 ha in 2013 and is likely to decline further to 0.08 ha by 2035.^3^ With the strong declining trend in per capita arable land and water availability, food security of the growing population which is projected to reach 1.44 billion by 2020, 1.51 billion by 2030, and 1.64–1.74 billion by 2050 is a cause of concern.^1^ Thus, agricultural land is under ever‐growing constraints to sustain agronomic productivity for feeding the ever increasing population in India. This scenario is further exacerbated by the rapidly increasing brick kilns in India which mine agriculturally productive top soil for the preparation of bricks.^4^ India is the second largest brick producer (≈250 billion bricks) after China with an annual growth rate of 2–5%.^5^ Over the millennia, going back to the Indus Valley Civilization, brick kilns have used productive soil and severely hindered the soil ecosystem services.^4^, ^6^ As much as 65% of total bricks are produced from the Gangetic plains comprising the states of Assam, Bihar, Haryana, Punjab, Uttar Pradesh (UP), and West Bengal,^4^ the region known for its fertile soil. Brick preparations involves removal of agriculturally productive top soil, that is, soil rich in clay and organic matter contents.^4^, ^5^ Along with degradation of agriculturally productive land, the production of fired bricks also causes emissions for greenhouse gases (GHGs) and other pollutants.^7^ Yet, the emission of carbon dioxide (CO_2_) and other GHGs has not been quantified adequately in relation to the brick making process in India. Thus, the objective of this study is to assess the effects of brick making in India on: (i) the annual CO_2_ emission, and (ii) agricultural production.

The data on SOC concentration of preburned brick soil are presented in **Table** **1**. On an average, each preburned brick weighs 1.8 kg, and has SOC concentration of 0.51–2.25% with a mean of 1.30%. At 95% confidence interval mean SOC concentration ranged from 1.24 to 1.36% (Table 1). We have used this value of SOC concentration (1.24–1.36%) for all computational purposes to address the issue of uncertainties associated with the estimation of SOC concentration and extrapolation. In comparison, concentration of SOC in burned bricks is 0 (zero) suggesting that all of the SOC was combusted and emitted as CO_2_ during the brick firing process. Therefore, burning of each brick (1800 g) leads to the loss of 22.32–24.48 g of C (an equivalent of 82–90 g of CO_2_). The SOC concentration varies from 0.2 to 2.7% in diverse geographical regions of India.^8^ As the range of distribution of SOC in preburned soil (range: 1.74, min: 0.51% and max: 2.25%) is similar to the reported data on SOC concentration in India, it is logical and justifiable to use the presently estimated SOC value in the Indian context. The present annual production of burned bricks in India is 250 billion.^4^, ^9^ Further, the size of bricks is almost consistent in India and, therefore, the data on SOC loss during the brick making process can be extrapolated to national scale.^5^ Hence, the amount of SOC lost through the firing process from 250 billion bricks in India is 5.58–6.12 Tg (Teragram, 1 Tg = 10^12^ g) which is equivalent to annual emission of 20.48–22.46 Tg of CO_2_ (1 C = 3.67 CO_2_). Indian brick industry consumes 15–20 Tg of coal annually, and it is the third largest consumer of coal in the country after power plants and the steel industry.^10^ Average CO_2_ emission in India due to combustion of coal during firing of the kiln was estimated at 80.7 kg CO_2_ per thousand bricks.^11^ Therefore, 250 billion bricks that are produced annually in India releases 20.18 Tg CO_2_ per annum. Together clay burning and coal combustion releases 40.65–42.64 Tg CO_2_ per annum in India, the latter is ≈3% of the gross CO_2_ emission from India.^12^ Total CO_2_ emission from brick making would be more than the value presented because our study did not incorporated dung and crop residue burnt during fired brick preparation due to lack of reliable data. Data presented reveal the magnitude of annual CO_2_ emission from the fired‐brick industry is alarming. At the present rate of C sequestration potential of Indian forests (≈4 Mg C ha^−1^ yr^−1^),^13^ ≈3 Mha afforestation is required per annum to offset the emission from the fired‐brick industries, which is an unfeasible option in a land‐scarce country. Nevertheless, rehabilitation of degraded lands to accelerate carbon sequestration is a viable option to offset emission from fired brick industry and other anthropogenic activities. Therefore, some of the 114–147 Mha of degraded land in India^14^ may be immediately rehabilitated through promoting traditional agroforestry or plantation systems. Agroforestry practices such as the fertilizer tree (fast‐growing nitrogen‐fixing) systems have been known to improve SOC build‐up in depleted soils.^15^ The soil carbon sequestration potential (0.5–0.8 Mg C ha^−1^ yr^−1^) of traditional agroforestry systems in India is higher than that of agricultural systems such as rice‐paddy and comparable to that of single‐species tree‐crop systems of rubber, areca, and coconut.^13^, ^16^ Promotion of agroforestry (*Piper betle* agroforestry) and plantations (e.g., rubber, bamboo, etc.) in those lands may significantly improve SOC stock in depleted soils in India.^13^ Rehabilitating abandoned brick kilns through bamboo plantation development in villages of Kotwa and Rahimabad of Allahabad, India over a time period of seven years (1996–2003) enhanced SOC sequestration from almost 0 (in 1996) to 0.7 (in 2003) Mg ha^−1^ yr^−1^ in the surface soil.^17^ Therefore, adoption of improved management systems can rehabilitate depleted brick kiln soil while accelerating soil carbon sequestration.

**Table 1:** Summary statistics for soil organic carbon (%) distribution in Barak Valley, North East India

Mean	1.30
Standard error	0.03
Median	1.32
Mode	1.25
Range	1.74
Minimum	0.51
Maximum	2.25
Count	400
Confidence level (95.0%)	0.06

The land area used by a brick kiln in India ranges from 1.0 to 9.0 ha with a mean value of 4.84 ha per unit (**Table** **2**). Currently, there are 140 000 brick kilns that produce ≈250 billion bricks annually with a growth rate of 2–5% annum^−1^.^4^ Extrapolation to the national scale indicates the total land area of 0.7 Mha (4.84 ha × 140,000 brick units) in India is currently under brick kilns. The overall urban population in India increased from 217 to 377 million during 1991 to 2011. The numbers of towns and cities have also increased from 3768 to 7951 during this period.^18^ Building construction in India is estimated to grow at a rate of 6.6% year^−1^ during the period 2005–2030.^19^ The number of building stock is expected to multiply five times during this period^5^ resulting into immense pressure on agricultural land for production of more bricks to fulfill increase in the demand for the building materials. Therefore, with an annual increase of 2%, more than 1 Mha of land area will be under brick kilns by 2030. Brick kilns not only degrade soil quality but also affects quality and quantity of agricultural productions.^20^, ^21^ Results from the on‐farm experimental plots revealed annual production of rice, mustard, and potato under abandoned brick kiln was 62, 91, and 67% less than that under control site (**Table** **3**). Tukey's test revealed the agricultural production of rice (*p* = 0.005), mustard (*p* = 0.042), and potato (*p* = 0.008) under abandoned brick kiln site was significantly different from adjacent managed agricultural site at 95% confidence level. Soils of the abandoned brick production sites are inherently low in fertility^20^ and fail to support higher crop yields even under fertilizer treatment. Studies on plant growth and productivity of three main vegetables *Brassica oleracea*, *Phaseolus vulgaris*, and *Solanum melongena* cultivated in the vicinity of the brick kiln area of the Panzan village, Budgam, India exhibited deterioration of the nutritional quality of the vegetables.^21^ Brick kiln impacts soil quality through degradation of physical, chemical, and biological properties^20^, ^22^ and may also jeopardize India's food security through declining quantity and quality of the food grains produced. Therefore, currently agricultural land of 0.7 Mha degraded by brick kilns cannot produce comparable crop yield with that under managed agricultural land (Table 3) and thus strongly aggravate the issue of food security. The annual average growth rate of the agriculture sector was 5% between 2004–2005 and 2007–2008, but decreased to 3% between 2008–2009 and 2013–2014.^3^ Moreover, agricultural and cultivable land has also declined from 185 Mha in 1980–1981 to 182 Mha in 2012–2013^23^ due to increase of agricultural lands for nonagricultural uses. Decline in prime agricultural land due to establishment of unregulated brick industries poses additional stress on agricultural growth.^6^ Therefore, the pressure emanating from brick kiln expansion and its degradation effect on soil health may exaggerate yield fluctuations, reduce nutritional quality of food (grains and milk) produced, and also have an adverse effect on livelihood of the farmers.

**Table 2:** Summary statistics for area under brick production unit in Barak Valley, North East India

Parameter	Area [ha]
Mean	4.837
Standard error	0.2356
Median	5
Range	8
Minimum	1
Maximum	9
Count	80
Confidence level (95.0%)	0.469

**Table 3:** Comparison of agricultural production under abandoned brick kiln and adjacent agricultural site in Barak Valley, North East India

Production [Mg ha^−1^ yr^−1^]	Brick kiln abandoned site (A)	Managed agricultural site (B)	% less yield in A over B
Rice (*Oryza sativa*)	1.6(0.2)a)	4.2(0.08)b)	62
Mustard (*Brassica nigra*)	0.1(0.01)a)	1.1(0.02)b)	91
Potato (*Solanum tuberosum*)	2.2(0.05)a)	6.8(0.09)b)	68

^a,b)^ Values within parentheses are standard errors of means. Different letters refers to significant differences between values in different rows. A: brick kiln abandoned site; B: Managed agricultural site.

In summary, the data presented indicate the positive feedback of the C‐emitting fired‐brick industries in accelerating the climate change phenomenon while degrading soil quality and jeopardizing food security. Currently, 0.7 Mha of land under brick industry needs to be rehabilitated through improved management systems. With a soil carbon sequestration rate of 0.5–0.8 Mg C ha^−1^ yr^−1^ under improved management systems, rehabilitation of 0.7 Mha land can sequester ≈0.5 Tg carbon annually. With sequestration of atmospheric CO_2_ being on global agenda since the initiation of “4 per thousand” at COP21 in 2015 and followed up at the COP22 in Marrakech, there is a strong need to restore the depleted soils. In addition to offsetting the gaseous emissions, protecting top soil of agro ecosystems is also essential to advancing food and nutritional security of rapidly increasing population of South Asia. The scientists, engineers, and policy makers in India, at national and regional level, must identify strategies of procuring alternate construction material without jeopardizing the prime agricultural land.

## Experimental Section

**Figure 1:**
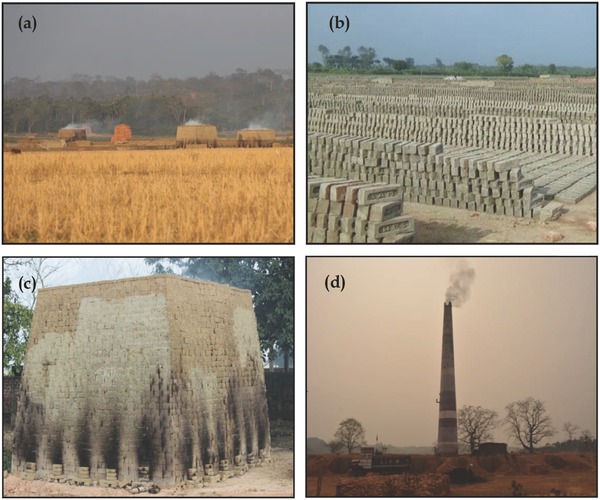
a) Traditional brick kilns on agricultural land, b) sun drying of clay bricks, c) square‐shaped brick burning unit, and d) gaseous emission from the brick kiln.

Soil samples were collected from fired‐brick making locations in North East India (NEI) for the purpose of estimating the emission of CO_2_ from the brick making process. There are two types of fired‐brick processes in NEI: (i) “square‐shaped without chimney” called “bangla bhattas” (**Figure** **1**a–c), and (ii) “conical‐shaped with a chimney” called “civil bhattas” (Figure 1d). The former produces ≈1 million bricks annually and engages 80–100 laborers per unit, whereas, the latter is a traditional fired‐brick production system with an annual production of ≈0.1 million bricks per unit and aims to fulfill individual or local needs. To achieve the desired research goals, soils from 80 fired‐brick industry (50 from civil bhattas and 30 from bangla bhattas) were collected from different locations in the Barak Valley (24°08′ to 25°08′N latitude and 92°15′ to 93°15′E longitude), with annual rainfall of ≈3200 mm. Soils of the Barak Valley are deep, the A horizon is 10–15 cm thick and has silty clay loam or clay loam texture.^24^ A detail of the soil physical and chemical properties of Barak Valley is presented in **Table** **4**. The dominant soil is classified as the Barak series, which is fine, mixed, hyperthermic family of Aeric Endoaquepts, which is the equivalent of Inceptisols in the USDA classification.^25^ According to the World Reference Base for Soil Resources classification and correlation system the soils correlate with Cambisols.^26^ From each brick industry, soil samples were collected randomly from five preburned and five burned bricks. Selection criteria for preburned and burned bricks were to represent freshly prepared clay bricks and freshly burnt bricks. Therefore, soil samples were assessed for soil organic carbon (SOC) from 400 preburned and 400 burned bricks. Soil samples from preburned bricks were collected to estimate how much SOC in clay bricks is burnt during the firing process. Similarly, samples from burned bricks were collected to estimate how much SOC in clay bricks is retained after the firing process. Prior to obtaining soil samples, the selected bricks were weighed to estimate the C content (%). Preburned and burned bricks were ground and ball‐milled, and sieved through 100 µm mesh for assessment of SOC concentration by the wet combustion method.^27^, ^28^ The conversion of SOC to CO_2_ was done following the procedure of IPCC.^29^ The area occupied by each industrial unit was also assessed through official records and interview with the industry owner during field surveys. To explore whether the fired brick industry also affects agricultural production of rice (*Oryza sativa*), mustard (*Brassica nigra*), and potato (*Solanum tuberosum*), on‐farm fertilizer experimental data under rain fed condition were utilized which include triplicate treatments from (a) control site (300–500 m away from abandon brick kiln), and (b) 6‐year‐old abandon brick kiln site. Each plot measured 5 m × 5 m in size and plots were established at a distance of 1 m from each other. Nitrogen, phosphorus, and potassium fertilizer (130:100:60) was used in the form of urea, single super phosphate, and muriate of potash in both the sites for all the crops. Fertilizer experimental plot was developed in 2014 in a randomized block design and continued till 2016. Statistical significance of the effects of brick kiln abandoned site and adjacent agricultural site on yield was assessed at *P* < 0.05 using Tukey's test.

**Table 4:** Soil physical and chemical properties of Barak Valley, North East India

Properties	Value
Sand [%]	4.4
Silt [%]	62.6
Clay [%]	33
pH (1:2.5 H_2_O)	5.3 (0.05)a)
Organic carbon [g kg^−1^]	1.08 (0.08)
Total nitrogen [g kg^−1^]	1.8 (0.3)
Ave phosphorus [me kg^−1^]	0.68 (0.07)
Ca^2+^ [cmol (+) kg^−1^]	4.0 (0.04)
Na^+^ [cmol (+) kg^−1^]	0.61 (0.05)
K^+^ [cmol (+) kg^−1^]	11.1 (0.7)
CEC	8.43 (0.21)
Base saturation [%]	70

^a)^ Standard error of the mean; Data Source: refs. 24, 30, 31.

## Conflict of Interest

The authors declare no conflict of interest.
